# Solution-processed Sb_2_Se_3_ photocathodes under Se-rich conditions and their photoelectrochemical properties†

**DOI:** 10.1039/d3ra07023a

**Published:** 2024-01-03

**Authors:** Hui Jin Jin, Chaeyong Seong, Gyu Wan Choi, Ji-Youn Seo, Min-Kyu Son

**Affiliations:** a Nano Convergence Materials Center, Emerging Materials R&D Division, Korea Institute of Ceramic Engineering & Technology (KICET) Jinju 52851 Republic of Korea minkyu.son@kicet.re.kr; b Department of Nano Fusion Technology, Pusan National University Busan 46241 Republic of Korea j-y.seo@pusan.ac.kr; c Department of Materials Science and Engineering, Korea University Seoul 02841 Republic of Korea

## Abstract

In this study, selenium (Se)-rich antimony selenide (Sb_2_Se_3_) films were fabricated by applying a solution process with the solvents ethylenediamine and 2-mercaptoethanol to optimize the photoelectrochemical (PEC) performance of the Sb_2_Se_3_ photocathode. Various antimony (Sb)–Se precursor solutions with different molar ratios of Sb and Se (Sb : Se = 1 : 1.5, 1 : 3, 1 : 4.5, 1 : 7.5, and 1 : 9) were prepared to attain Se-rich fabrication conditions. As a result, the Se-rich Sb_2_Se_3_ films fabricated using the Sb–Se precursor solution with a molar ratio of Sb : Se = 1 : 7.5 exhibited an improved PEC performance, compared to the stoichiometric Sb_2_Se_3_ film. The charge transport was improved by the abundant Se element and thin selenium oxide (Se_2_O_3_) layer in the Se-rich Sb_2_Se_3_ film, resulting in a decrease in Se vacancies and substitutional defects. Moreover, the light utilization in the long wavelength region above 800 nm was enhanced by the light-trapping effect because of the nanowire structure in the Se-rich Sb_2_Se_3_ film. Hence, the optimal Se-rich Sb_2_Se_3_ photocathodes showed an improved photocurrent density of −0.24 mA cm^−2^ at the hydrogen evolution reaction potential that was three times higher than that of the stoichiometric Sb_2_Se_3_ photocathodes (−0.08 mA cm^−2^).

## Abstract 2

Photoelectrochemical (PEC) characteristics of Se-rich Sb_2_Se_3_ photocathodes controlling the Sb–Se precursor solution with different molar ratio in the solution process: enhanced PEC performance by the improved charge transport and light utilization.

## Graphical abstract

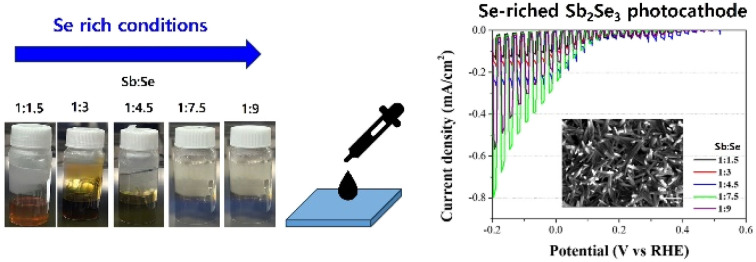

## Introduction

Novel materials with p-type characteristics have been intensively researched to produce an efficient and durable photocathode in a photoelectrochemical (PEC) water splitting system.^1–5^ Antimony selenide (Sb_2_Se_3_) has received much attention as a promising candidate material for this purpose,^6–10^ because it has several attractive features for an efficient, durable, and economical PEC water splitting system. Sb_2_Se_3_ is a p-type semiconductor with a suitable band position for the hydrogen evolution reaction.^11–13^ Moreover, Sb_2_Se_3_ has a small band gap (1.1–1.2 eV) with a high light absorption coefficient.^14–17^ Hence, it is theoretically possible to produce a high current density up to approximately −40 mA cm^−2^, thereby achieving a high solar-to-hydrogen conversion efficiency.^12,18^ Sb_2_Se_3_ also has an excellent electron mobility (16.9 cm^−2^ V^−1^ s^−1^) that is much higher than the mobility of cuprous oxide, which is a representative photocathode material.^19^ Furthermore, Sb_2_Se_3_ is immune to photocorrosion in an aqueous solution, particularly under acidic conditions.^20–22^ In addition, it is inexpensive because the cost of the main element (Sb) in the molecule is relatively lower than that of other expensive light absorbers, such as indium (In), gallium (Ga), and molybdenum (Mo).^12,19^

The morphological characteristics of the Sb_2_Se_3_ film are a key parameter affecting the PEC performance of Sb_2_Se_3_ photocathodes. The morphological characteristics can be easily controlled by adjusting the fabrication process. A compact and pinhole-free Sb_2_Se_3_ photocathode can be fabricated by controlling the cooling speed in a close space sublimation method.^20^ The fast-cooling process is effective for suppressing the growth kinetics of Sb_2_Se_3_, resulting in a smooth Sb_2_Se_3_ film with few pinholes. The different solvents used in the solution process also determine the morphology of the Sb_2_Se_3_ photocathodes. A combined solution of ethylenediamine (EDA) and 2-mercaptoethanol (2-MER) is advantageous for fabricating a thin and compact Sb_2_Se_3_ film, whereas, a solution with thioglycolic acid (TGA) and ethanolamine (EA) is suitable to form the nanowire/rod-based Sb_2_Se_3_ films.^23^ In the solution process using the EDA/2-MER solvent, the nanostructured Sb_2_Se_3_ film can be obtained by controlling the concentration of Se/Sb in the precursor solution.^24,25^ On the other hand, the length and diameter of nanowire/rod can be controlled by changing the concentration of TGA/EA and the spin coating iteration.^26,27^ In this method, a planar Sb_2_Se_3_ film with few pinholes is beneficial for improving the charge transport in the Sb_2_Se_3_ photocathode, whereas a nanowire/rod-based Sb_2_Se_3_ film is favorable to enhancing light utilization. Therefore, it is crucial to control the morphology of the Sb_2_Se_3_ film by adjusting the fabrication process parameters to optimize the PEC performance of the Sb_2_Se_3_ photocathode.

The compositional characteristics of Sb_2_Se_3_ films are a potential factor affecting on the PEC performance of Sb_2_Se_3_ photocathodes, as well as their morphological characteristics. Recently, it has been reported that Se-rich Sb_2_Se_3_ films enhance the efficiency of Sb_2_Se_3_-based photovoltaic cells because such films have fewer Se vacancies and substitutional defects acting as charge recombination centers.^28,29^ However, a reduction in efficiency has been observed in the excessively Se-rich Sb_2_Se_3_ photovoltaic cells. Thus, these considerations may be applied in photocathodes for PEC water splitting because their basic operation principle is similar to that of photovoltaic cells. Hence, optimal Se-rich Sb_2_Se_3_ films should be prepared to attain Sb_2_Se_3_ photocathodes with a high PEC performance.

Therefore, in this study, Sb_2_Se_3_ photocathodes were fabricated using a solution process with EDA and 2-MER as solvents as they allow for easy control of the film composition. The concentration of Se in the precursor solution with a fixed concentration of Sb was gradually increased to obtain the Se-rich Sb_2_Se_3_ photocathode. Morphological, optical, compositional, and electrochemical analyses were carried out to investigate the effect of the Se-rich Sb_2_Se_3_ photocathode on the PEC performance depending on the concentration of Se. As a result, the Se-rich Sb_2_Se_3_ photocathode fabricated using the precursor solution with the optimal concentration of Se showed an improved PEC performance because of the enhanced charge transport, as well as the improved light utilization as a result of the nanostructured morphology.

## Experimental details

### Preparation of the precursor solution

A precursor solution based on the EDA and 2-MER solvents was prepared using a published method with further modifications.^24^ Briefly, the precursor solvent was prepared by adding EDA (Sigma-Aldrich, 99.5%, 8 mL) and 2-MER (Sigma-Aldrich, 99%, 2 mL) to a vial. The mixture was left to stand with the molecular sieves (Sigma-Aldrich, bead size of 1.6–2.6 mm) for 1 h to remove moisture. Then, Sb–Se precursor solutions with different molar ratios were obtained by mixing Sb (Sigma-Aldrich, 99.5%) and Se (Sigma-Aldrich, 99.5%) powders in the prepared solvent. To prepare the Se-rich solution, the amount of Se was varied, while that of Sb was fixed. The solution was stirred overnight at 70 °C and 500 rpm on a hot plate for a thorough dissolution. The prepared Sb–Se precursor solution was filtered using a PTFE membrane filter (0.45 μm) to remove the remaining Sb/Se powders before film deposition.

### Fabrication of the Sb_2_Se_3_ photocathode

Fluorine doped tin oxide (FTO) coated glass (Sigma-Aldrich, thickness of 2.2 mm, surface resistivity of 7 Ω sq^−1^) was prepared as a substrate for the Sb_2_Se_3_ photocathodes. Before film deposition, the FTO substrate was cleaned by sequential ultrasonication processes in acetone, ethanol, and distilled water for 10 min, respectively. Additional UV treatment was carried out for 30 min to remove the remaining residues on the FTO substrate. The prepared Sb–Se precursor solution was spin coated on the cleaned FTO substrate at 2500 rpm for 25 s. The number of spin coating was fixed at two, to prevent the thickness from affecting the PEC performance of the Sb_2_Se_3_ photocathode. The coated samples were dried at 180 °C for 3 min on a hot plate after the first spin coating process. The Sb_2_Se_3_ photocathodes were completed by annealing at 250 °C for 20 min on the hot plate after the second spin coating process. All deposition processes were carried out under the inert N_2_ gas in a glovebox.

### Material characterization

X-ray diffraction (XRD) analysis was carried out to determine the crystallinity of the Sb_2_Se_3_ photocathodes using a high-resolution XRD system (D8 ADVANCE, Bruker) with a Cu-Kα source (*λ* = 1.54060 Å) in the 2*θ* range of 10°–70°. The morphologies of the solution processed Sb_2_Se_3_ photocathodes were analyzed using a field emission scanning electron microscope (FE-SEM, S8000, TESCAN) equipped with an energy dispersive X-ray (EDX, Oxford Instruments) analyzer. The optical properties of the Sb_2_Se_3_ photocathodes were acquired by a UV-VIS-NIR photospectroscopy (V-670, JASCO). X-ray photoelectron spectroscopy (XPS) was carried out using an XPS system with automated surface analysis (NEXSA, Thermo Fisher Scientific) to investigate the surface properties of the Sb_2_Se_3_ photocathodes including their components and bonding characteristics.

### Electrochemical and photoelectrochemical characterization

The PEC performance of the Sb_2_Se_3_ photocathodes was measured using a standard three-electrode configuration system consisting of a solution processed Sb_2_Se_3_ photocathode as a working electrode, a Pt wire as a counter electrode, and an Ag/AgCl reference electrode in saturated KCl. The Sb_2_Se_3_ photocathode was masked with opaque epoxy (Loctite, EA E-60HP) to determine the active area of the Sb_2_Se_3_ photocathode before the PEC measurement. Linear sweep voltammetry (LSV) measurements were carried out in a 0.1 M H_2_SO_4_ aqueous solution (pH 1) with a scan rate of 10 mV s^−1^ under one sun illumination (AM 1.5 G, 100 mW cm^−2^) from a solar simulator (XES-50S2, SAN-EI ELECTRIC). The measured potential was converted into a reversible hydrogen electrode (RHE) scale, using the following equation.*E*_RHE_ = *E*_Ag/AgCl_ + 0.059 × pH + 0.197

Chronoamperometry (CA) measurements were carried out in the same electrolyte (0.1 M H_2_SO_4_) biased at 0 V *versus* RHE under chopped one sun illumination to determine the stability of the Sb_2_Se_3_ photocathodes. The CA measurement is a typical method to evaluate the stability of the photoelectrode in the PEC water splitting system because it is an intuitive technique to recognize the activity of the photoelectrode on the water reduction reaction.^30^ A decrease in the photocurrent density during the CA measurement often indicates the corrosion of the photoelectrode in the aqueous solution.

To examine the charge transport capability of the Sb_2_Se_3_ photocathodes, the electrochemical impedance spectroscopy (EIS) measurement was conducted in a 0.1 M H_2_SO_4_ aqueous solution under one sun illumination biased at the hydrogen evolution reaction potential (0 V *versus* RHE). It was carried out at frequencies from 1 MHz to 100 mHz by applying a sinusoidal potential perturbation of 10 mV. All data of PEC and EIS measurements were acquired by a potentiostat (SP-200, BioLogic Science Instruments).

## Results and discussion

**Fig. 1:**
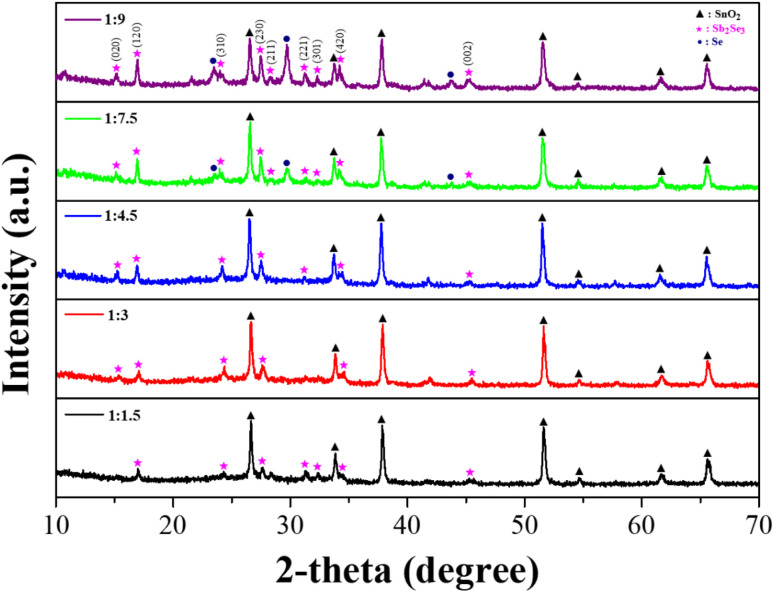
XRD patterns of solution processed Sb_2_Se_3_ film with different molar ratios of Sb and Se; 1 : 1.5 (black), 1 : 3 (red), 1 : 4.5 (blue), 1 : 7.5 (green) and 1 : 9 (purple).

Different amounts of Sb and Se powders were dissolved in the Sb–Se precursor solution based on the EDA and 2-MER mixed solvent. To control the Se-rich conditions, the amount of Sb powder was fixed, while the amount of Se powder was slightly increased, according to the molar ratio of Sb and Se; (Sb : Se) = 1 : 1.5, 1 : 3, 1 : 4.5, 1 : 7.5, and 1 : 9, respectively. Fig. 1 shows the XRD patterns of the solution processed Sb_2_Se_3_ films using different Sb–Se precursor solutions. All films exhibited XRD patterns indexed to crystalline Sb_2_Se_3_ (JCPDS 15-0861) and XRD patterns indexed to SnO_2_ (JCPDS 46-1088) from the FTO substrate.^31–33^ Moreover, no oxide phases such as Sb_2_O_3_ were observed in the XRD patterns. This indicates that the deposited film on the FTO substrate was a well-crystallized Sb_2_Se_3_ film. On the other hand, the intensity of the peaks related to the (120) and (230) orientations became stronger, as the amount of Se powder was increased. This is clear evidence of the formation of an Sb_2_Se_3_ nanowire film with a preferred orientation of (001), which was horizontally laid on the substrate.^24,34^ Interestingly, the XRD pattern indexed to elemental Se (JCPDS 06-0362) appeared in the solution processed Sb_2_Se_3_ film with a molar ratio of Sb : Se = 1 : 7.5.^35^ Moreover, its intensity was remarkably increased in the solution processed Sb_2_Se_3_ film with a molar ratio of Sb : Se = 1 : 9. This result is likely due to the excessive Se components in the Sb_2_Se_3_ film. It was also confirmed by the Se/Sb atomic ratio detected by the EDX system (Fig. S1 and Table S1†). The Se/Sb atomic ratio was gradually increased along with the increase in the amount of Se powders, indicating the formation of a Se-rich Sb_2_Se_3_ film. Therefore, it was demonstrated that an Sb–Se precursor solution with excessive Se powders is favorable for obtaining the solution processed Se-rich Sb_2_Se_3_ film.

**Fig. 2:**
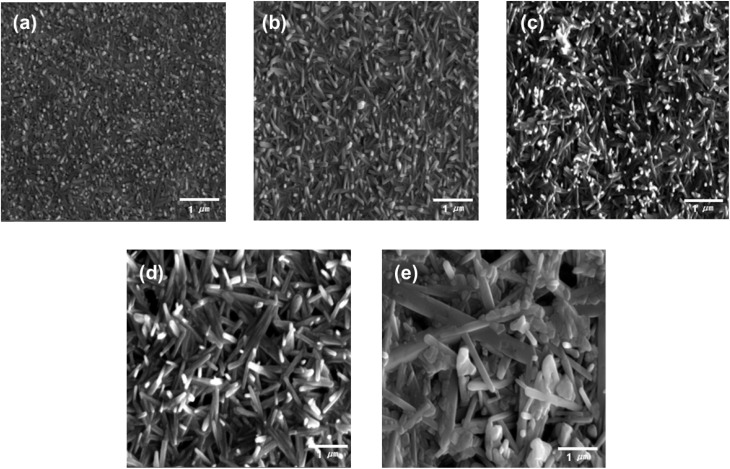
Top-view FE-SEM images of the solution processed Sb_2_Se_3_ films with different molar ratios of Sb and Se. (a) Sb : Se = 1 : 1.5, (b) Sb : Se = 1 : 3, (c) Sb : Se = 1 : 4.5, (d) Sb : Se = 1 : 7.5, and (e) Sb : Se = 1 : 9.

Fig. 2 presents the FE-SEM images of the solution processed Sb_2_Se_3_ film with different molar ratios of Sb and Se. The stoichiometric Sb_2_Se_3_ film (Sb : Se = 1 : 1.5) was almost planar as it was formed by the agglomeration of short column-like Sb_2_Se_3_ grains. However, the morphology of the Sb_2_Se_3_ film was slightly changed into a nanowire structure as the amount of Se powder in the Sb–Se precursor solution was increased. Moreover, the length and diameter of the Sb_2_Se_3_ nanowire were continuously increased along with the increment in the amount of Se. In the precursor solution with the excessive Se amount, the longer 1D [Sb_4_Se_7_]^2−^ chain bonding along the (001) axis is predominantly formed by the complete reaction with the sufficient Se chains and Sb ions.^24^ This accelerates the formation of longer Sb_2_Se_3_ nanowires with a larger diameter. This finding agrees with the XRD results (Fig. 1). Finally, using the precursor solution with excessive Se (Sb : Se = 1 : 9), an irregular nanowire structured Sb_2_Se_3_ film was obtained including a large nanowire with a diameter of approximately 400 nm and many voids. The numerous voids in the Sb_2_Se_3_ film are likely due to the high viscosity of the precursor solution under the high Se condition. The morphological changes of the Sb_2_Se_3_ film, particularly the formation of the Sb_2_Se_3_ nanowire, would affect the PEC performance of the Sb_2_Se_3_ photocathodes because they provide a sufficiently large surface area for the water reduction reaction, direct routes for the efficient charge transport, as well as advantageous structures for the enhanced light utilization.

**Fig. 3:**
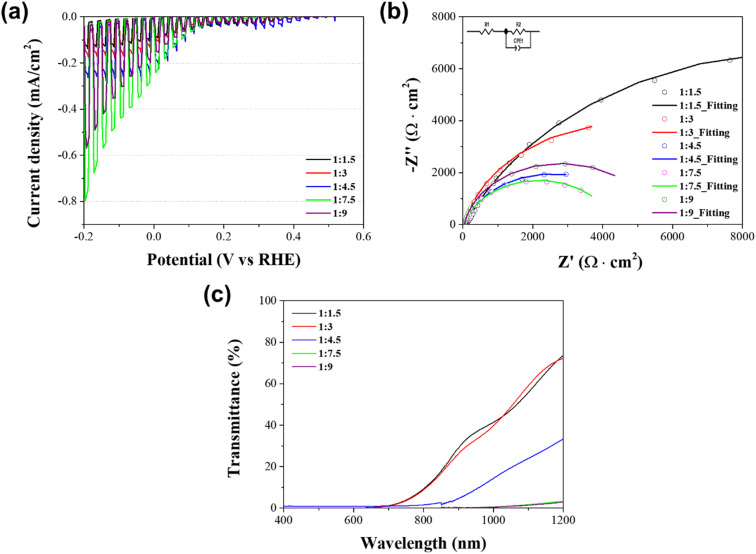
(a) Current density–potential curves in a 0.1 M H_2_SO_4_ aqueous solution (pH 1) under chopped one sun illumination, (b) Nyquist plots and fitting curves biased at 0 V *versus* RHE under continuous one sun illumination, (c) transmission spectra of Sb_2_Se_3_ photocathodes with different molar ratios of Sb : Se; 1 : 1.5 (black), 1 : 3 (red), 1 : 4.5 (blue), 1 : 7.5 (green) and 1 : 9 (purple).

Fig. 3a shows the PEC performance of the solution processed Sb_2_Se_3_ photocathodes with different molar ratios of Sb : Se in a strongly acidic aqueous solution (0.1 M H_2_SO_4_) under chopped one sun illumination. The PEC performance, particularly the photocurrent density, gradually improved as the Se concentration was increased. The highest photocurrent density (−0.24 mA cm^−2^ at 0 V *versus* RHE) corresponded to the Sb_2_Se_3_ photocathodes with a molar ratio of Sb : Se = 1 : 7.5. By contrast, the photocurrent density slightly decreased in the Sb_2_Se_3_ photocathodes with a molar ratio of Sb : Se = 1 : 9, although the concentration of Se was further increased. Thus, the PEC performance was related to the variation of the charge transport capability and light utilization in the Sb_2_Se_3_ photocathode, resulting from the morphological changes of the Sb_2_Se_3_ film.

To examine the charge transport capability of the Sb_2_Se_3_ photocathodes, the EIS measurement was carried out under one sun illumination. Fig. 3b illustrates the Nyquist plots of the solution processed Sb_2_Se_3_ photocathodes with different molar ratios of Sb : Se, acquired by the EIS measurement. The plots were fitted based on the equivalent circuit connecting the sheet resistance and the impedance component in series, as shown in the inset of Fig. 3b. In general, the sheet resistance (*R*_s_) is related to the substrate of the electrode.^36^ Hence, it was almost similar in all Sb_2_Se_3_ photocathodes because the same FTO substrate was used. On the other hand, the impedance component was composed of the interface resistance between the electrolyte and the Sb_2_Se_3_ photocathode (*R*_ct_) and the constant phase element. Table 1 shows the *R*_ct_ values of the Sb_2_Se_3_ photocathodes with different molar ratios of Sb : Se, extracted from the semi-circle in the Nyquist plot. It was gradually decreased along with the increment in the Se concentration. Finally, the smallest *R*_ct_ value (4255 Ω), which was approximately one-fifth of the *R*_ct_ (19 624 Ω) in the stoichiometric Sb_2_Se_3_ photocathode with a molar ratio of Sb : Se = 1 : 1.5, corresponded to the Sb_2_Se_3_ photocathode with a molar ratio of Sb : Se = 1 : 7.5. This result indicates that the charge transport in the photocathode/electrolyte interface was enhanced in the Se-rich Sb_2_Se_3_ photocathode and was mainly attributed to the intrinsic characteristics of the Se-rich Sb_2_Se_3_ film. The longer nanowire structure in the Se-rich Sb_2_Se_3_ film (Fig. 1) facilitated the improved charge transport in the Sb_2_Se_3_ photocathode by providing a larger surface area for the water reduction reaction as well as effective charge transport routes. In addition, the fewer Se vacancies in the Se-rich Sb_2_Se_3_ substantially decreased the charge recombination centers, resulting in the improved charge transport of the Sb_2_Se_3_ photocathodes.^28,29^ However, the excessively Se-rich Sb_2_Se_3_ photocathode with a molar ratio of Sb : Se = 1.9 showed a decreased PEC performance, which is likely due to the many voids in the irregular nanowire structure (Fig. 2e) that act as charge recombination sites. The trend of the *R*_ct_ values agrees with the PEC performance of the solution processed Sb_2_Se_3_ photocathodes with different molar ratios of Sb : Se (Fig. 3a).

**Table 1:** Sheet resistance (*R*_s_) and electrolyte/photocathode interface resistance (*R*_ct_) of the Sb_2_Se_3_ photocathodes with different molar ratios of Sb : Se

Sb : Se	1 : 1.5	1 : 3	1 : 4.5	1 : 7.5	1 : 9
*R*_s_	62.19	46.12	44.55	50.47	44.37
*R*_ct_	19 624	9411	5032	4255	5507

The improved PEC performance of the Se-rich Sb_2_Se_3_ photocathode was also confirmed by the optical properties of solution processed Sb_2_Se_3_ photocathodes. Fig. 3c displays the transmittance of the Sb_2_Se_3_ photocathodes with different molar ratios of Sb : Se. The transmittance in the wavelength region above 800 nm decreased as the concentration of Se increased. This indicates that the light absorption in the long wavelength region is enhanced along with the increment in the Se concentration. It is a good agreement with the absorbance spectra of Sb_2_Se_3_ photocathodes (Fig. S2†). The thickness of deposited Sb_2_Se_3_ films was almost similar (approximately 0.7–0.8 μm, Fig. S3†), except for the Sb_2_Se_3_ film with a molar ratio of Sb : Se = 1 : 9 (1.5 μm) because of the high viscosity of the precursor solution. Thus, it is possible to exclude the thickness of Sb_2_Se_3_ as a major parameter affecting the light absorption. Therefore, the obtained result is mainly attributed to the morphological change from a planar Sb_2_Se_3_ film to a nanowire structured film, which improves the light utilization in the long wavelength region because of the light trapping effect.^24,37^ This is one of the main reasons why the Se-rich Sb_2_Se_3_ photocathode exhibited an improved PEC performance compared to the stoichiometric Sb_2_Se_3_ photocathode, together with an enhanced charge transport. Based on the optical properties, the estimated band gap of the Se-rich Sb_2_Se_3_ photocathode fabricated using the Sb–Se precursor solution with excessive Se powders was 1.14–1.15 eV (Table S2†), which is a traditional band gap region of Sb_2_Se_3_ films.^38^

**Fig. 4:**
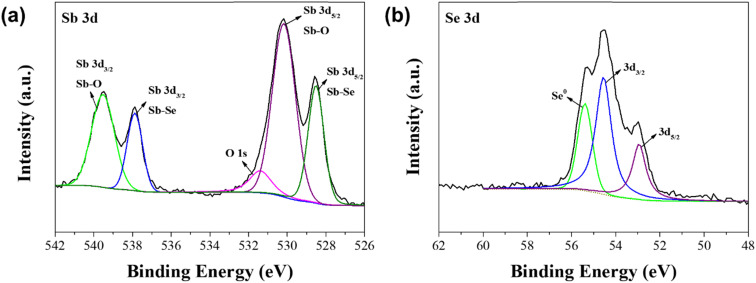
XPS spectra of the Sb_2_Se_3_ photocathode with a molar ratio of Sb : Se = 1 : 7.5; (a) Sb 3d core level and (b) Se 3d core level.

XPS measurements were carried out to further analyze the elemental composition of solution processed Sb_2_Se_3_ photocathodes with different molar ratios of Sb : Se. Fig. 4 displays the XRD spectra of the Sb_2_Se_3_ photocathode fabricated by using the Sb–Se precursor solution with a molar ratio of Sb : Se = 1 : 7.5, which showed the best PEC performance. In the spectrum of Sb 3d core level (Fig. 4a), two peaks appeared at 537.9 eV and 528.5 eV, which correspond to the bonding of Sb and Se from the Sb_2_Se_3_ film.^39–41^ Two additional peaks appeared at 539.5 eV and 530.2 eV. These peaks are ascribed to the Sb–O bond from the Sb_2_O_3_ film on the surface of the Sb_2_Se_3_ film.^39,40^ These peaks were not observed in the XRD spectrum (Fig. 1) because they were the result of the slight oxidation of Sb at the surface of the crystallites. In the spectrum of Se 3d core level (Fig. 4b), three peaks appeared at 52.9, 54.6, and 55.4 eV. The two peaks located at 52.9 eV and 54.6 eV are ascribed to Se^2−^ in the Sb_2_Se_3_ film, whereas the peak located at 55.4 eV is ascribed to the elemental Se.^39,40^ The intensity of the peak located at 55.4 eV began to appear when the Sb : Se molar ratio was above 1 : 7.5 (Fig. S4†). This means that the Se-concentrated precursor solution is essential to fabricate the Se-rich Sb_2_Se_3_ photocathode, using the solution process. It is thought that the Sb_2_O_3_ thin film on the surface and the abundance of Se in the Se-rich Sb_2_Se_3_ photocathode facilitate the passivation of Se vacancies and substitutional defects in the Sb_2_Se_3_ film, resulting in the reduced charge recombination centers.^29^ Hence, the PEC performance of the Se-rich Sb_2_Se_3_ photocathode was considerably improved, compared to that of the stoichiometric Sb_2_Se_3_ photocathode.

**Fig. 5:**
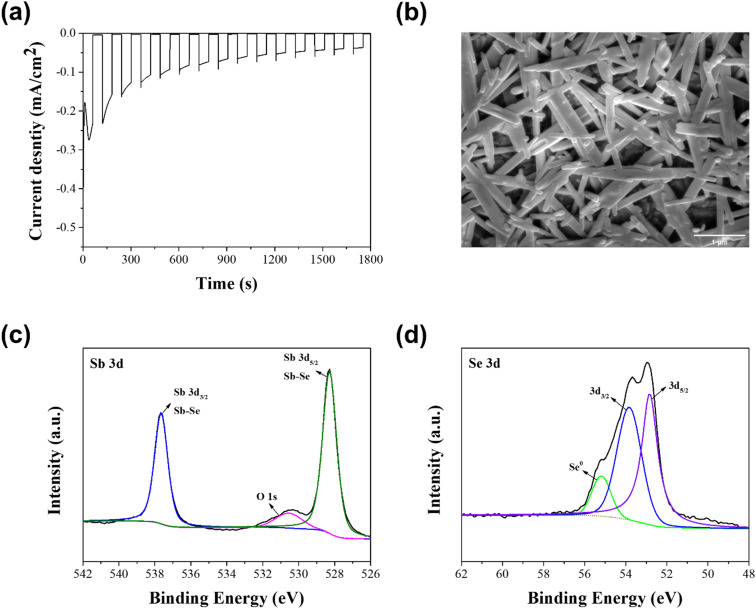
(a) Current-density of the Se-rich Sb_2_Se_3_ photocathode during the stability test in a 0.1 M H_2_SO_4_ aqueous solution (pH 1) under chopped one sun illumination, (b) top-view FE-SEM image and (c, d) XPS spectra of the Se-rich Sb_2_Se_3_ photocathode after stability test for 30 min. The Sb_2_Se_3_ photocathode with a molar ratio of Sb : Se = 1 : 7.5 was used for the stability test.

Photocathode stability is also a critical issue for the practical PEC water splitting, as well as the PEC performance. Hence, a stability test was carried out in a 0.1 M H_2_SO_4_ aqueous solution under the continuous PEC operation, using the Se-rich Sb_2_Se_3_ photocathode with a molar ratio of Sb : Se = 1 : 7.5, which showed the best PEC performance. As shown Fig. 5a, the photocurrent density gradually decreased during the PEC operation for 30 min. This outcome was mainly attributed to the morphological and compositional changes of the Se-rich Sb_2_Se_3_ photocathodes. Fig. 5b shows a top-view FE-SEM image of the Se-rich Sb_2_Se_3_ photocathode with a molar ratio of Sb : Se = 1 : 7.5 after the stability test was conducted for 30 min. The diameter of the Sb_2_Se_3_ nanowire was slightly enlarged compared to that before the stability test (Fig. 2d). Hence, many voids between the Sb_2_Se_3_ nanowires were observed after the stability test. These voids play a role as charge recombination sites, resulting in a decreased PEC performance. In addition, a compositional change was revealed by the XPS spectra of the Se-rich Sb_2_Se_3_ photocathode after the stability test (Fig. 5c and d). The two peaks related to the Sb–O bond from the Sb_2_O_3_ disappeared in the spectrum of Sb 3d core level (Fig. 5c), whereas the peak ascribed to the elemental Se remained in the spectrum of Se 3d core level (Fig. 5d). This result indicates that the Sb_2_O_3_ thin film on the surface of the Se-rich Sb_2_Se_3_ photocathode vanishes because of the dissolution of the Se-rich Sb_2_Se_3_ film during the PEC operation, which likely induces the exposure of Se vacancies or substitutional defects in the Sb_2_Se_3_ film to the electrolyte, thereby diminishing the PEC performance.

Compared to the previously reported PEC performance of Sb_2_Se_3_ photocathodes (Table S3†), the photocurrent density is extremely low because our Se-rich Sb_2_Se_3_ photocathode consists of only the Sb_2_Se_3_ film, without any modifications such as a back contact layer, n-type overlayer, or hydrogen evolution reaction catalyst. However, it is quite competitive, compared to bare or Au underlayered Sb_2_Se_3_ photocathodes in the reported literatures. In addition, it is possible to enhance the stability of Se-rich Sb_2_Se_3_ photocathodes by introducing a TiO_2_ protection layer. Therefore, works on the additional modifications using novel and low-cost materials are underway to further enhance the PEC performance and stability of Se-rich Sb_2_Se_3_ photocathodes, based on the optimal solution process conditions reported in this work.

## Conclusion

It is well-known that the Se-rich Sb_2_Se_3_ thin films are advantageous for improving the efficiency of Sb_2_Se_3_ based solar cells. Inspired by this fact, in this work, we fabricated a Se-rich Sb_2_Se_3_ photocathode by introducing the solution process, using an Sb–Se precursor solution based on the EDA and 2-MER solvents for improving the PEC performance of the Sb_2_Se_3_ photocathode. To control the characteristics of the Se-rich Sb_2_Se_3_ photocathode, various Sb–Se precursor solutions with different molar ratios of Sb : Se were prepared by adjusting the amount of Se while using a fixed amount of Sb. As a result, the charge transport in the Sb_2_Se_3_ photocathode was gradually improved in accordance with the increment in the Se amount. This outcome is likely due to the reduced Se vacancies and defects in the Sb_2_Se_3_ photocathode, resulting from the passivation effect by the Se elements and the Sb_2_O_3_ thin film on the surface of the photocathode. In addition, the morphology of the Sb_2_Se_3_ photocathode changed from a planar structure to a nanowire structure by increasing the Se amount in the Sb–Se precursor solution. This nanowire morphology is also beneficial for improving the PEC performance because the light utilization in the long wavelength region was enhanced by the light trapping effect. However, the excessively Se-rich Sb_2_Se_3_ photocathode showed a lowered PEC performance because of the morphological defects in the irregular nanowire structure with many voids. Therefore, it is crucial to optimize the concentration of Se in the Sb–Se precursor solution to obtain Se-rich Sb_2_Se_3_ photocathodes with an enhanced PEC performance. Finally, the Se-rich Sb_2_Se_3_ photocathode with a molar ratio of Sb : Se = 1 : 7.5 exhibited the highest PEC performance with a photocurrent density of −0.24 mA cm^−2^ at 0 V *versus* RHE, which was three times higher than that of the stoichiometric Sb_2_Se_3_ photocathode.

## Conflicts of interest

There are no conflicts to declare.

## Supplementary Material

RA-014-D3RA07023A-s001
