# Supplementary material for: Solution-processed Sb2Se3 photocathodes under Se-rich conditions and their photoelectrochemical properties
Source: RSC Adv. 2024 Jan 3;14(1):59–66. doi: 10.1039/d3ra07023a (PMC10762725; doi:10.1039/d3ra07023a)
Supplement: RA-014-D3RA07023A-s001 [file RA-014-D3RA07023A-s001.pdf]

Electronic Supplementary Material (ESI) for RSC Advances.
This journal is © The Royal Society of Chemistry 2023

Supplementary Information

Solution-processed Sb_2Se_3 Photocathodes under Se-rich Conditions and Their Photoelectrochemical Properties

Hui Jin Jin[1,2], Chaeyong Seong[1,3], Gyu Wan Choi[1,2], Ji-Youn Seo[2*], Min-Kyu Son[1*]

[1]Nano Convergence Materials Center, Emerging Materials R&D Division, Korea Institute of Ceramic Engineering & Technology (KICET), Jinju 52851, Republic of Korea
[2]Department of Nano Fusion Technology, Pusan National University, Busan 46241, Republic of Korea
[3]Department of Materials Science and Engineering, Korea University, Seoul 02841, Republic of Korea

*E-mail: j-y.seo@pusan.ac.kr, minkyu.son@kicet.re.kr

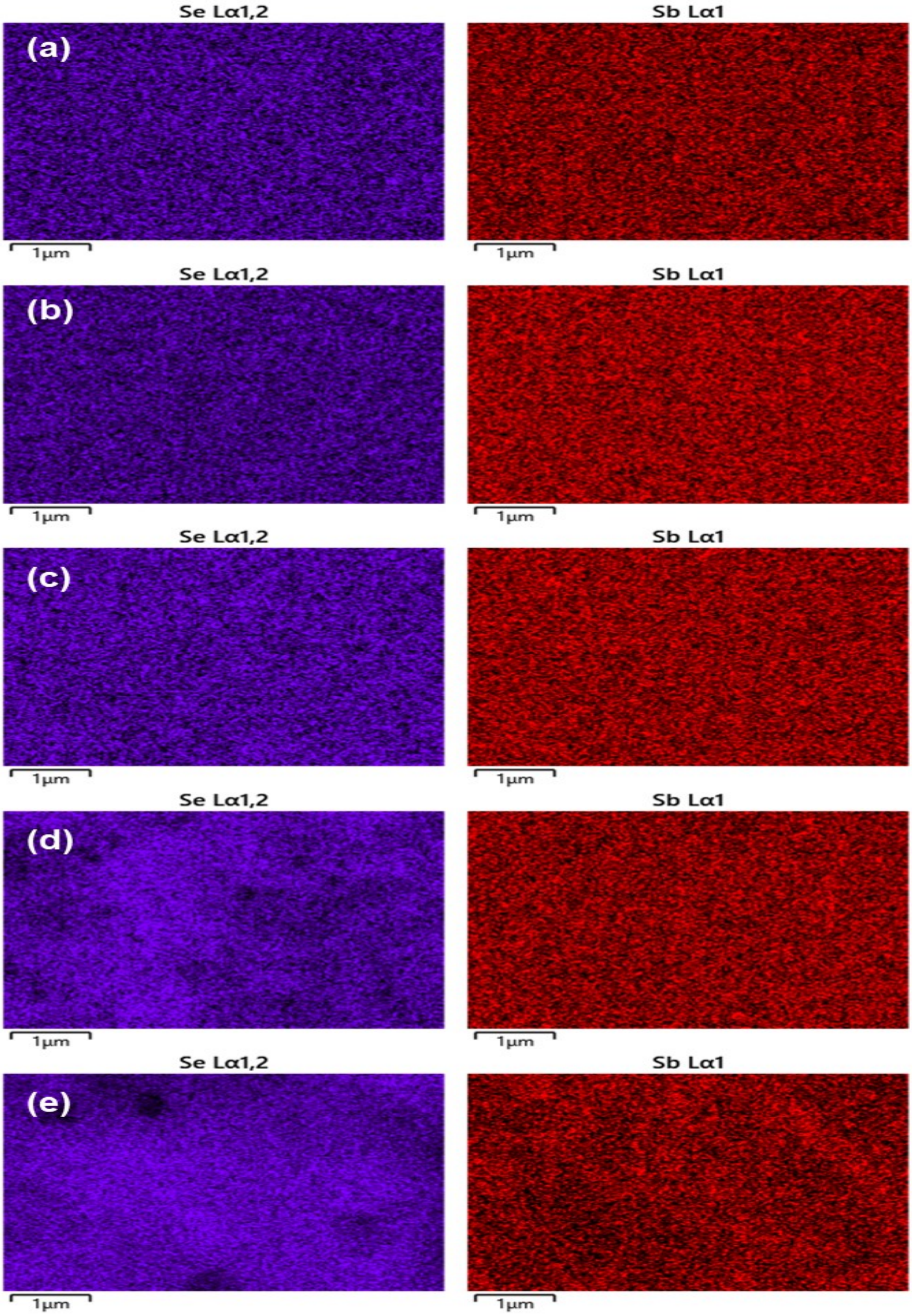

Figure S1. EDX elemental map showing proportional distribution of Sb and Se. a) Sb:Se = 1:1.5, b) Sb:Se = 1:3, c) Sb:Se = 1:4.5, d) Sb:Se = 1:7.5, e) Sb:Se = 1:9.

Table S1. Sb and Se atomic ratios detected by EDX system.

Sb:Se	Se	Sb
1:1.5	29.63	4.64
1:3	34.64	7.20
1:4.5	37.49	8.72
1:7.5	50.48	7.16
1:9	71.50	10.52

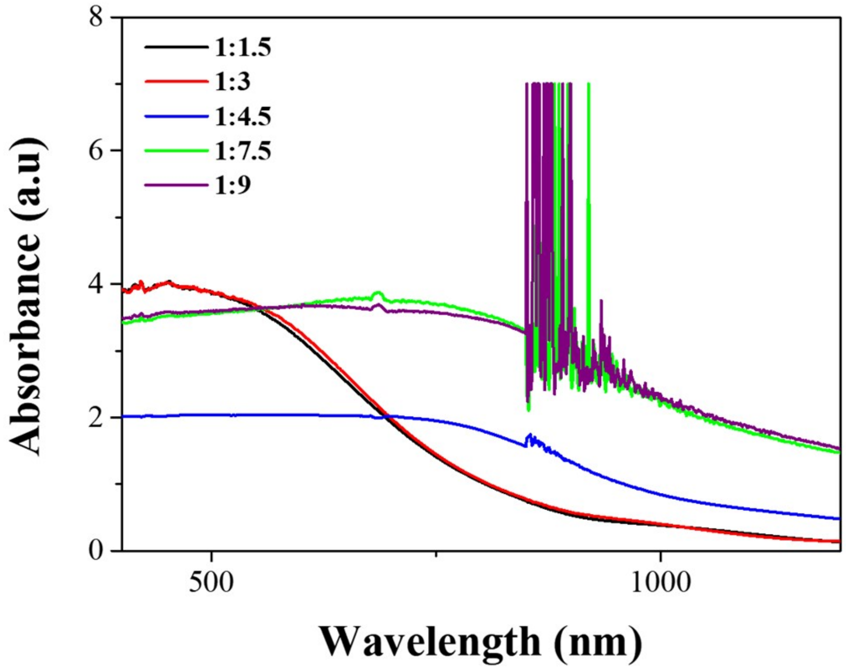

Figure S2. Absorbance spectra of Sb_2Se_3 photocathodes with different molar ratios of Sb:Se; 1:1.5 (black), 1:3 (red), 1:4.5 (blue), 1:7.5 (green) and 1:9 (purple).

Table S2. Estimated band gap of Sb_2Se_3 photocathodes with different molar ratios of Sb:Se from Tauc-plot.

Sb:Se	1:1.5	1:3	1:4.5	1:7.5	1:9
Band gap (eV)	1.73	1.71	1.25	1.15	1.14

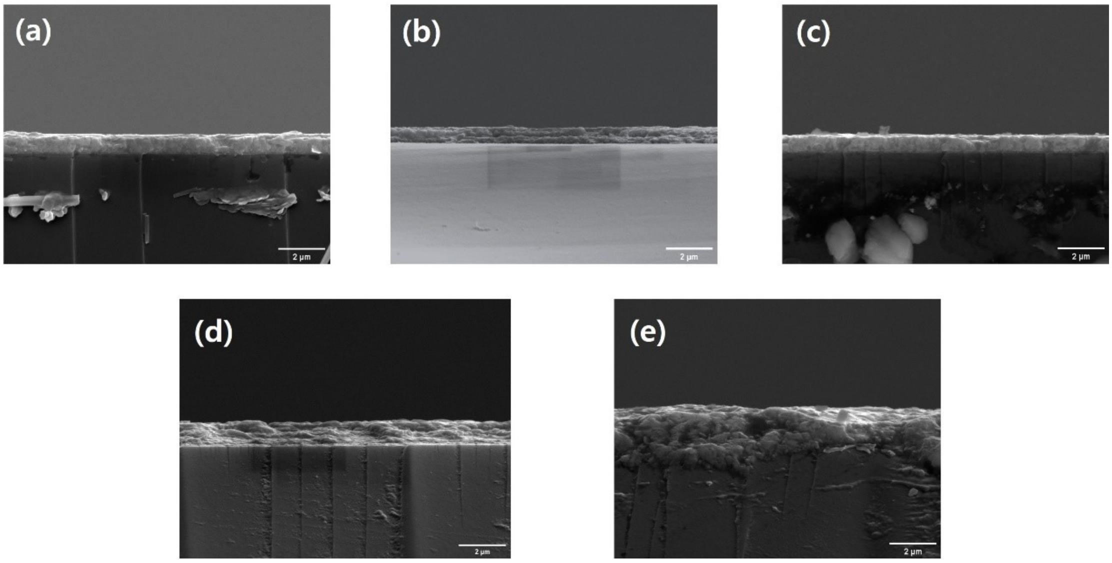

Figure S3. Cross-view FE-SEM images of the solution processed Sb_2Se_3 films with different molar ratios of Sb and Se. (a) Sb:Se = 1:1.5, (b) Sb:Se = 1:3, (c) Sb:Se = 1:4.5, (d) Sb:Se = 1:7.5, and (e) Sb:Se = 1:9.

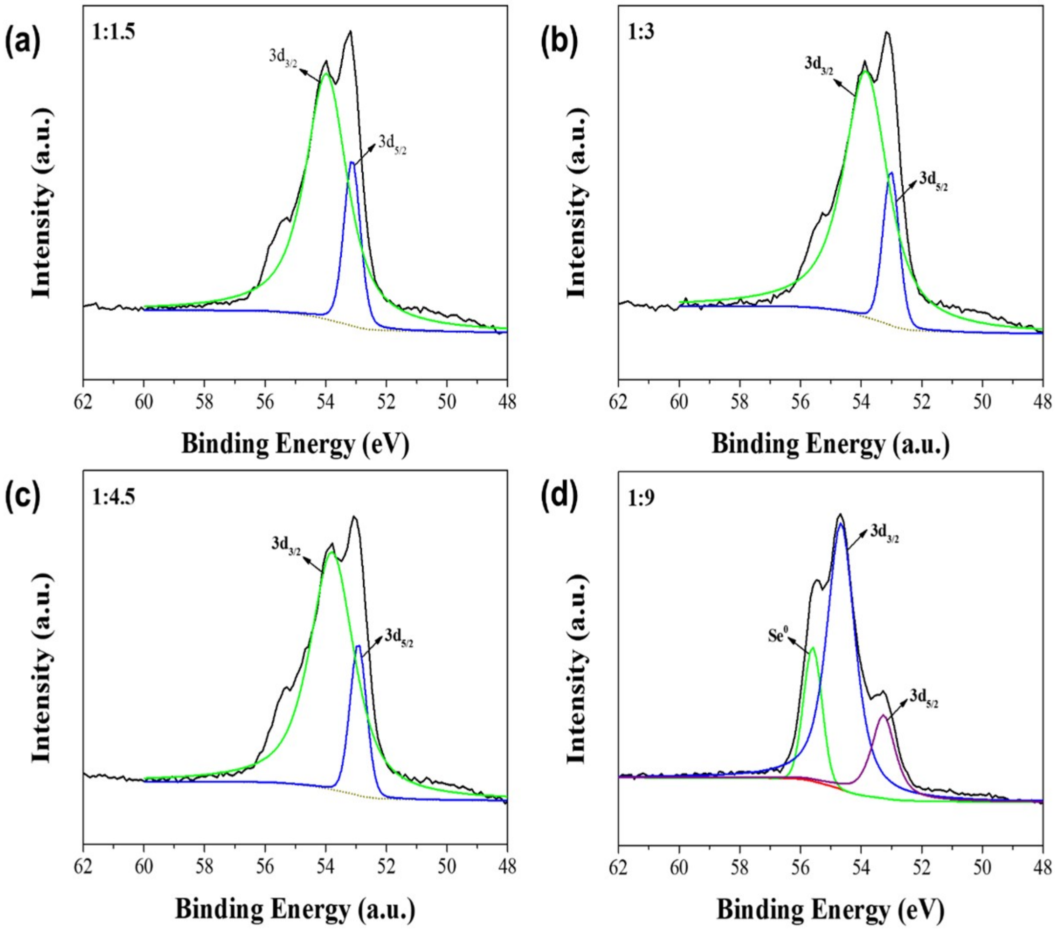

Figure S4. XPS spectra in Se 3d of Sb_2Se_3 photocathode according to Sb:Se molar ratios. (a) Sb:Se = 1:1.5, (b) Sb:Se = 1:3, (c) Sb:Se = 1:4.5, (d) Sb:Se = 1:7.5, and (e) Sb:Se = 1:9.

Table S3. Photoelectrochemical performances of Sb_2Se_3 photocathode in other literatures and our work. (SnS_2 : Tin sulfide, Fh : Ferrihydrite, MoS_x : Molybdenum sulfide, RuO_x : Ruthenium oxide, OL : Organic layer, CdS : Cadmium sulfide, Cu:NiO_x : Cu-doped nickel oxide, C_{60} : Fullerene)

Fabrication method	Composition (Substrate: FTO)	Onset Potential (V *vs* RHE)	Photocurrent density (mA/cm^2 at 0 V *vs* RHE)	Electrolyte	*Ref.*
Vapor transport deposition	Sb_2Se_3	0.3	-0.02	0.5 M Na_2SO_4 (pH 7)	10
	Sb_2e_3/SnS_2/Fh	0.3	-1.0		
Electrodeposition	Sb_2Se_3	0.2	-0.1	Na_2SO_4 (pH 6.5)	8
	Sb_2e_3/Pt	0.2	-1.4		
Electrodeposition of Sb /Selenization	Au/Sb_2Se_3	·	·	1 M H_2SO_4 (pH 0)	21
	Au/Sb_2Se_3/MoS_x	0.12	-6		
	Au/Sb_2Se_3/MoS_x (Sulfurization)	0.25	-14		
Sputtering of Sb /Selenization	Mo/Sb_2Se_3/CdS/TiO_2/Pt	0.54	-35.7	0.5 M H_2SO_4	16
Sputtering of Sb /Selenization	Mo/Sb_2Se_3/TiO_2/Pt	0.3	-20	1 M H_2SO_4	18
Close space sublimation	Au/$Sb_2Se_3/TiO_2/RuO_x$	0.4	-30	H_2SO_4 (pH 1)	20
	Au/Sb_2Se_3/CdS/TiO_2/Pt	0.5	-18.5		
Close space sublimation	Au/Sb_2Se_3/OL/TiO_2/Pt	0.5	-35	H_2SO_4 (pH 1)	7
Solution process	Au/Sb_2Se_3/TiO_2/Pt	0.3	-30	0.1 M H_2SO_4 (pH 1)	23
Solution process	Au/Sb_2Se_3/CdS/TiO_2/Pt	0.5	-14	H_2SO_4 (pH 1)	24
Solution process	Au/Sb_2Se_3	0.5	-0.32	0.5 M H_2SO_4	26
	Au/Sb_2Se_3/TiO_2/Pt	> 0.4	-2.5		
Solution process	Sb_2Se_3/TiO_2/Pt	0.3	-10	H_2SO_4 (pH 1)	11
	Cu:$NiO_x/Sb_2Se_3/TiO_2$/Pt	0.34	-17.5		
Solution process	Au/Sb_2Se_3/TiO_2/Pt	0.35	-12.5	0.1 M H_2SO_4 (pH 1)	17
Solution process	Au/$Sb_2Se_3/TiO_2/C_{60}$/Pt	0.4	-17	H_2SO_4 (pH 1)	6
Solution process	Au/Sb_2Se_3	0.3	-0.18	0.5 M H_2SO_4	9
	Au/Sb_2Se_3/TiO_2/Pt	0.3	-11.3		
Solution process	Sb_2Se_3	0.45	-0.24	0.1 M H_2SO_4 (pH 1)	Our work
